# Development of Solid Organ Transplantation in Syria

**Published:** 2011-02-01

**Authors:** B. Saeed

**Affiliations:** *Kidney Hospital, Kidney Transplant Department, Damascus, Syria*

## INTRODUCTION

Syria is a country with a population of 23 millions. The first kidney transplant in Syria was performed in 1979 at Harasta Hospital in Damascus. The donor was a living relative; the recipient received simple immunosuppressants comprising of azathioprine and steroids as the transplantation took place prior to the cyclosporine era. However, the renal allograft remained functional for more than 25 years. The transplant surgeon was an eminent urologist, Dr. Maher Al-Houssami, who 25 years later, became the Syrian minister of health and played a major role in setting the national guidelines that regulated the legal and medical aspects of organ donation and transplantation in Syria. More importantly, he was a fearless leader who worked hard in both words and deeds for banning commercialism in this field.

**ORGAN TRANSPLANTATION IN SYRIA**

Kidney is still the only solid organ that is transplanted in Syria. Bone marrow transplantation started in 2008 at Tichreen Hospital in Damascus. In 1990, heart transplantation was performed for few patients at Tichreen Hospital in Damascus but unfortunately, the program was stopped. Liver, pancreas, lung, and intestine transplantation have never been performed in Syria. Cornea transplantation has been and is still performed predominantly in private hospitals using “purchased” corneas. However, nearly 1500 corneas were transplanted in public sector at Eye Surgical Hospital in Damascus using donated corneas till 2004 when for lack of offered corneas, number of cornea transplant was dramatically diminished in this center. Consequently, many patients with corneal blindness are still waiting for sight to be restored [1].

**RENAL FAILURE IN SYRIA**

The estimated incidence of end-stage renal disease (ESRD) in Syria is 100 pmp [1]. Out of a Syrian population of 23 millions, the estimated number of new ESRD cases is around 2300 every year. The prevalence of ESRD patients undergoing dialysis in Syria has substantially increased by 75% over the last five years. In 2005, 2860 patients with ESRD were on dialysis which makes the ESRD prevalence to be around 143 pmp for a population of 20 millions at that time [2].

The peritoneal dialysis (PD) modality is very unpopular and grossly underused in Syria; in 2005, less than 4% of patients on dialysis were receiving continuous ambulatory PD (CAPD) [2]. This underuse of CAPD in Syria, as in some other parts of the world [3, 4] is partly due to physician bias. Other reasons in Syria are low socio-economic status of certain patients, lack of skilled personnel which results in high rate of infection, recurrent peritonitis, and other technical problems.

In 2009, nearly 5000 patients with ESRD were on dialysis which makes the estimated prevalence of ESRD patients undergoing dialysis in 2009 to be around 217 pmp. Unfortunately, we do not have data on the percentage of patients with ESRD who receive dialysis in Syria, although we know that the acceptance rate for renal replacement therapy is less than that reported in the developed world where it ranges from 61% to 99% [5]**. **The 3-year survival rate of Syrian dialysis population has been estimated in 2005 to be up to 64% [2].

**PRE-EMPTIVE KIDNEY TRANSPLANTATION PRACTICE IN SYRIA**

Pre-emptive kidney transplantation in Syria is still restricted to a few patients. However, there is currently a tendency to apply it for an increasing number of patients as most transplant teams in the country have realized its lower costs and favorable outcomes since the entire kidney transplants performed are currently being harvested from living donors only.

**KIDNEY TRANSPLANTATION IN SYRIA**

**Figure 1:**
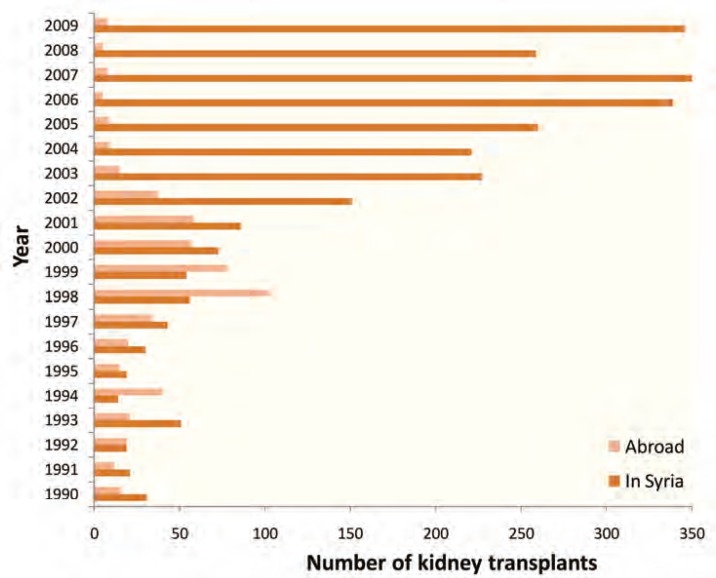
Trend in the total number of kidney transplants done inside *vs* outside Syria

During the 1980s, the practice of kidney transplantation in Syria was exclusively limited to living related donors and conducted at main public hospitals in Damascus (Tichreen and Al-Mouwassat Hospitals) totaling to 20–30 per year. During the 1990s, kidney transplant rate did slightly improve but remained sluggish around 2 pmp per year till the late 1990s when the total transplants exceeded 50 per year. In 2001, Kidney Hospital was established in Damascus and the total transplants was increased to 86 in 2001 and 151 in 2002 (Fig 1). Although the rate has increased to 7 pmp in 2002, the need for kidney transplant in Syria was very far from being met and the supply of transplantable kidneys remained fairly insufficient and was responding only to approximately 10% of the demand which was estimated to be around 75 kidneys pmp per year [6]. This big gap between the demand and supply of kidneys, together with the persisting low rate of kidney transplantation, has led to a severe shortage of kidney supplies. Consequently, a substantial increase in the rate of kidney transplantation performed on Syrian nationals abroad was observed, from 8% of all kidney transplantations in 1988 to 65% in 1998 when it reached its highest rate. However, it diminished again to 20%–45% in the early 2000s (Fig 1).

**NEW LAW AUTHORIZING DECEASED AND LIVING UNRELATED DONATION**

In November 2003, in response to the widening gap between demand and supply of kidneys and other organs, “law number 30” was enacted which recognized the concept of brain death and permitted transplantation from deceased unrelated donors with a national guidance on organ transplantation which stated that the donation act must be altruistic and between nationals to avoid transplant tourism. That legislation has been considered as a landmark in the history of organ transplantation in Syria [2]**.**

In November 2004, the Syrian Ministry of Health issued guidelines that regulated the legal and medical aspects of organ donation and transplantation, including the definition of death and brain death criteria, the consent for deceased organ donation, banning commercialism, and how to evaluate potential organ donors.

**CLIMBING KIDNEY TRANSPLANT RATE IN SYRIA**

As a result of the 2003 national Syrian legislation, the kidney transplant rate jumped from seven kidney transplants pmp in 2002 to 17 pmp in 2007. Subsequently, the rate of kidney transplantation performed on Syrian nationals abroad declined to less than 2% in 2007 (Fig 1). It is well known that the number of kidney transplants performed pmp correlates with the socioeconomic status of a country [6]. The 2007 kidney transplant rate in Syria (>17 pmp) was quiet above that of most developing countries where the kidney transplant rate ranges from 1 to 5 pmp with an average of 2 pmp in the Middle East and the Afro-Arab region [7].

Obviously, the increased rate of kidney transplant inside the country and the declined rate of abroad transplants were the two major positive impacts of the *law number 30*. However, the practice of kidney transplantation following this new law has been associated with some negative consequences such as the practice of “kidney selling” which, although prohibited by law, quickly became a common and readily available source of organs, and vendors have found ways to sell their kidneys through disreputable brokers especially in the private sector. This practice has raised ethical concerns regarding organ commercialism, exploitation of the poor, and undermining public trust in the transplant system [8].

**CHARACTERISTICS OF LIVING KIDNEY DONATION IN SYRIA**

**Figure 2:**
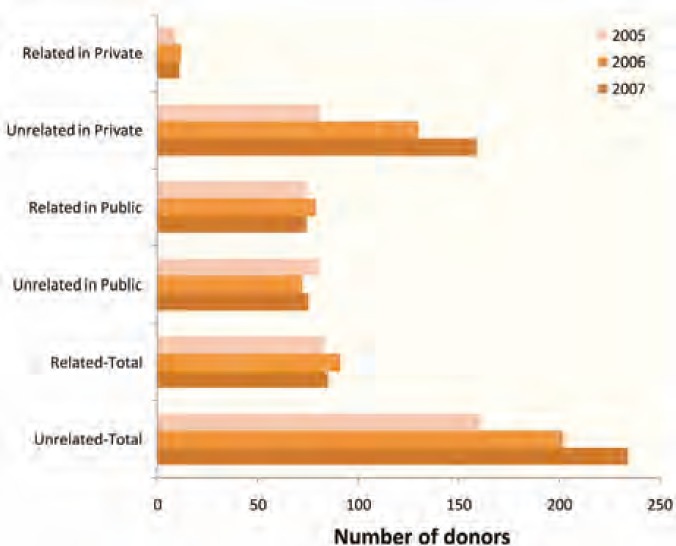
Number of related and unrelated kidney donors in Damascus public and private hospitals

Following the new law of 2003, the practice of kidney donation by unrelated living “volunteers” flourished in the private sector where a substantial increase of kidney transplant rate in this sector by more than 9-fold within five years (2002–2007) has been noticed [8]. By 2007, 54% of the 350 transplants in Syria were done in private hospitals where 92% of donors were unrelated as compared to 50% in public hospitals and 70 % of both sectors together [8]. The growing practice of unrelated donation in private sector has been in fact at the expense of decreasing the percentage of related donors to as little as 8% of all donors in private sector as compared to 50% in public sector (Fig 2). Meanwhile, the kidney transplant rate in public sector did not increase following the 2003 legislation, but rather declined to 8 pmp in 2007 as compared to 9.5 pmp in the same year in private sector. This indicates a steady trend toward expanding kidney transplant activities from unrelated donors in private sector and decreasing the rate of kidney transplant in public sector as the majority of potential related donors (71%) were reluctant to donate kidneys when kidneys could be bought from a non-related donor [8]! Reversing that trend was in fact one of the major concerns of health authorities not only because such a trend could lead to possible closure of public kidney transplant centers in which kidney vendors were not welcome but more importantly because of the practice of organ commercialism that was obviously inconsistent with the principles expressed in the World Health Assembly (WHA) 57.18 resolution on human organ and tissue transplantation of May 2004 [9], which called upon member states to “take measures to protect the poorest and vulnerable groups from transplant tourism and the sale of tissues and organs.”

**RESTRICTING KIDNEY TRANSPLANTATION TO PUBLIC SECTOR**

In January 2008, following a detailed discussion and exchange of thoughts and opinions on how to approach and offset this dilemma in order to take the necessary actions that would fulfill the direction of the aforementioned resolution, the government of Syria issued a pronouncement restricting kidney transplantation to the public sector with a new national regulatory oversight of transplantation practices in a way to make the donated organs as community resources and not to be marketed for financial gain. The ministry of health formed independent commissions of physicians, lawyers, and psychiatrists with a main task of interviewing both recipients and donors to see whether the essential conditions are met. The Transplantation Society (TTS) has commended the government of Syria for this pronouncement and consider it as an important testimony to fulfill the WHA resolution of 2004 and as a step toward ethical propriety that would have a global impact [8].

Since this 2008 Administrative Order was promulgated, a substantial increase of kidney transplant rate in public hospitals has been noticed and reached 110% by December 2009 as compared to 2007. In other words, the kidney transplant rate in public hospitals has been more than doubled within a period of less than two years (162 kidney transplants in 2007 *vs* 346 in 2009) so that in 2009, the public sector alone has performed almost the same number of kidney transplants as that of private and public together in 2007 (Fig 1). The expansion of kidney transplant centers in the public sector with the establishment of four new public transplant centers in the three largest cities in Syria (Al-Assad University Hospital in Damascus, Al-Watani Hospital in Homs, Ibn-Rochd Hospital in Aleppo, and Damascus Hospital in Damascus) has also marked this period. These new centers undoubtedly contributed to the above mentioned increase of kidney transplant rate in public sector.

**UNRELATED DONATION AND THE “INEVITABLE KIDNEY SELLING” PRACTICE**

One has to confess that, unfortunately the practice of “kidney selling,” although prohibited, has continued in public hospitals after the 2008 Administrative Order but seemingly to a lesser extent and mostly without the interference of middle men or brokers. The persistent practice of illegal “kidney selling” in public sector is being considered as a complicated problem which seems quiet difficult to be under control since obviously, very often it is hard for any committee that interviews donors and recipients to fully assure the absence of any sort of money exchange between donors and recipients. This practice has been—and still is—raising ethical concerns in Syria regarding organ commercialism, exploitation of the poor, and undermining public trust of the transplant system because Syria, like the rest of the world, is in a global struggle to combat organ commercialism according to the principles expressed in The Declaration of Istanbul [10], which was an international summit held in Istanbul in 2008 and convened by TTS and ISN with 152 participants from 78 countries including two participants from Syria; the meeting resulted in the “Declaration of Istanbul on Organ Trafficking and Transplant Tourism” which aims to halt these unethical activities and to foster safe and accountable practices that meet the needs of transplant recipients while protecting donors. Undoubtedly, the Declaration of Istanbul has created intense pressure on countries to address their transplantation practices and many illegal outlets for transplantation in destination countries such as China and Pakistan were shut down. The health authorities in Syria expressed discomfort and displeasure with the frequency of unrelated donation, however there is reluctance to abruptly discontinue this practice since such an action might lead to a sharp decline in kidney transplant rate in the country with all its grave consequences regarding the patients quality of life, availability of dialysis facilities, rebounding kidney transplantation abroad and transplant tourism.

**PERSPECTIVES OF ORGAN TRANSPLANTATION IN SYRIA**

In perspective, if we are targeting to perform kidney transplantation for 75% of our new patients with ESRD which are equivalent to 75 pmp per year (since the remaining 25% might not be good candidates for transplantation due to a variety of reasons including medical contraindications), this optimal rate of transplantation is quiet higher than what is presently being done which was 15 kidney transplants pmp in 2009 (346 kidney transplants for a population of 23 millions).Therefore, we could figure out that in 2009, only 20% of the estimated optimal need for kidney transplantation was met in our country. 

These results enable us to conclude that there is a marked disparity between the number of patients with ESRD and the number of patients who received a transplant in Syria. Such a disparity might keep growing if no proper actions are taken in the near future in order to reverse the curve and to narrow the gap between the supply and demand of kidneys in Syria.

A national deceased donation program is a viable option to address the widening gap between organ demand and availability; for instance, the increasing request for kidneys is not only due to the increased number of patients with ESRD waiting for kidney transplantation, but also to the fact that patients who previously would not have been considered for transplantation (*e.g.*, patients with diabetes, the elderly, and children) are now on the waiting lists. With this respect, the “law number 30” that recognized the concept of brain death and permitted deceased donor transplantation, has been seen as a major step for initiating a deceased organ donation program that has to be activated to lessen the burden of living donors and to enable a national self-sufficiency not only in kidneys but also in all other organs and tissues. This very important law has been preceded by another big stride in this regard which was the acceptance of the higher Islamic religious authorities in the country back in September 2001 on the principle of procurement of organs from cadavers providing consent is given by a first- or second-degree relative. Such a progress could only be achieved after several meetings which gathered religious authorities, legislators, lawyers, health care professionals, patients, and lay public. Unfortunately, seven years after the enactment of “law number 30,” yet there is no deceased donor program in Syria due to multiple reasons and obstacles but one thing is sure that deceased donation program in a country cannot take off if commercialism is going on!

**INITIATING A DECEASED DONATION PROGRAM IN SYRIA**

It is worth saying that from the legislative point of view, some of the major obstacles to initiate a national deceased donor program in Syria have been overcome by the “law number 30” that recognized the concept of brain death and permitted deceased organ donation, and also by the support of most religious commentators—both Muslim and Christian.

In November 2009, The Ministry of Health issued the law for initiating The Syrian National Center for Organ Transplantation. That was indeed another step forward, at least from the legislative point of view, but it also requires logistics, setup, and financial support by the government. Such a center is fundamental for the success of cadaveric donation program, as it supervises and coordinates the whole process of organ donation between the donating hospital and the transplant center, in addition to so many other functions like applying strategies to increase the awareness of the medical community and public at large to the importance of organ donation, and particularly, emphasizing ethics as the center is a nonprofit governmental agency. However, there are still several other obstacles that need to be properly tackled on the top of which is organ commercialism owing to the practice of living unrelated donor transplantation which although has fallen into disrepute, still remains the main source of kidneys in Syria, even for those patients who might have suitable living related donors.

Ignorance appears to be a major limiting factor inhibiting the initiation of cadaveric organ donation program in Syria as in many other developing countries [11]. Therefore, there is a need for a concerted and ongoing education campaign by the transplant community of both the health care professionals and the public to increase their awareness of the need for organ donation so as to change negative public attitudes and to gain societal acceptance. The success of this program definitely requires a high degree of public trust and acceptance. 

The indifferent attitude of health care professionals has also been identified as a major limiting factor to the initiation of cadaveric organ donation program, exactly as it has been pointed out in other developing countries [11, 12] and changing such attitudes should be given priority.

Lack of trained transplant coordinators is one of the important issues that have to be addressed before initiating a cadaveric donation program. Transplant coordination is still in its infancy in Syria. With an understanding of local social and cultural beliefs and sensitivity to the need and concerns of families, transplant coordinators could form a vital link between the community and the transplant team.

The program should not rely only on the enthusiasm of the transplant team without additional remuneration for the extra work performed. Adequate resources in terms of financial incentives are crucial, because deceased donor programs tend to be more expensive than living donor transplants and are constrained in countries where health resources are stretched to the limit [6].

Access to intensive care facilities is required to allow the ventilation of donors; therefore, the shortage of intensive care (ICU) beds can be a major limitation [12, 13]. More ICU beds are absolutely needed before a cadaveric donation program could be started. Moreover, the education and training of key personnel in the ICUs is of equal (if not more) importance than increasing number of ICU beds, because they are the ones who will identify potential donors, and then, they will ensure good donor maintenance in order to improve the quality of recovered organs.

One of the major obstacles to the initiation of deceased donation program in Syria is the lack of a defined waiting list of the transplant candidates and the lack of an estimate of the deceased donor potential.

National registry and data bank for all organ failure cases are still lacking in Syria; in the absence of such database, it is impossible to have precise ideas of the prevalence and incidence of organs failure which are substantial elements for profiling the national policy of organs transplantation.

Syria, like all other countries, must address its transplantation needs and strive for self-sufficiency in this field which should be considered as an objective for the government of Syria as stated in the WHA *Resolution 63.22* of May 2010 [14], where Member States are urged “to strengthen national and multinational authorities and/or capacities to provide oversight, organization and coordination of donation and transplantation activities, with special attention to maximizing donation from deceased donors.” The WHA endorsed the WHO Guiding Principles (GPs) on the transplantation of human cells, tissues and organs through *Resolution 63.22* which was signed by Syria; The GPs bring the maximization of deceased donation (number of donors and organs recovered and transplanted per donor) to the upfront and stress other important components of donation and transplantation, as donation being a voluntary and unpaid act and transparency as a safeguard. In fact, through these WHO GPs, WHO could assist Syria in the development of a legal and organizational framework for organ donation and transplantation in consistency with the Istanbul Declaration on organ trafficking and transplant tourism.

## CONCLUSION

Today, after more than three decades from the first kidney transplantation in Syria, although kidney transplant rate is higher than most Afro-Arab and Middle Eastern countries, all kidney transplants are still relying on living donors and there is no program of deceased donation in Syria. However, the recent law for initiating The Syrian National Center for Organ Transplantation and the expansion of kidney transplant centers in the public sector are two important initial steps in initiating a deceased organ donation program that has to be established to lessen the burden of living donors and to enable a national self-sufficiency not only in kidney but in all other organs and tissues.
